# Standard operating procedures for antibiotic therapy and the occurrence of acute kidney injury: a prospective, clinical, non-interventional, observational study

**DOI:** 10.1186/cc13918

**Published:** 2014-06-12

**Authors:** Irit Nachtigall, Sascha Tafelski, Karsten Günzel, Alexander Uhrig, Robert Powollik, Andrey Tamarkin, Klaus D Wernecke, Claudia Spies

**Affiliations:** 1 Department of Anaesthesiology and Intensive Care Medicine, Charité - Universitätsmedizin Berlin, Campus Charité Mitte and Campus Virchow-Klinikum, Augustenburger Platz 1, Berlin 13353, Germany; 2 Department of Internal Medicine, Division of Infectious Diseases and Pulmonary Medicine, Campus Charité Mitte, Berlin 10117, Germany; 3 Charité - Universitätsmedizin Berlin and SOSTANA GmbH, Berlin 10117, Germany

## Abstract

**Introduction:**

Acute kidney injury (AKI) occurs in 7% of hospitalized and 66% of Intensive Care Unit (ICU) patients. It increases mortality, hospital length of stay, and costs. The aim of this study was to investigate, whether there is an association between adherence to guidelines (standard operating procedures (SOP)) for potentially nephrotoxic antibiotics and the occurrence of AKI.

**Methods:**

This study was carried out as a prospective, clinical, non-interventional, observational study. Data collection was performed over a total of 170 days in three ICUs at Charité – Universitaetsmedizin Berlin. A total of 675 patients were included; 163 of these had therapy with vancomycin, gentamicin, or tobramycin; were >18 years; and treated in the ICU for >24 hours. Patients with an adherence to SOP >70% were classified into the high adherence group (HAG) and patients with an adherence of <70% into the low adherence group (LAG). AKI was defined according to RIFLE criteria. Adherence to SOPs was evaluated by retrospective expert audit. Development of AKI was compared between groups with exact Chi^2^-test and multivariate logistic regression analysis (two-sided *P* <0.05).

**Results:**

LAG consisted of 75 patients (46%) versus 88 HAG patients (54%). AKI occurred significantly more often in LAG with 36% versus 21% in HAG (*P* = 0.035). Basic characteristics were comparable, except an increased rate of soft tissue infections in LAG. Multivariate analysis revealed an odds ratio of 2.5-fold for LAG to develop AKI compared with HAG (95% confidence interval 1.195 to 5.124, *P* = 0.039).

**Conclusion:**

Low adherence to SOPs for potentially nephrotoxic antibiotics was associated with a higher occurrence of AKI.

**Trial registration:**

Current Controlled Trials ISRCTN54598675. Registered 17 August 2007.

## Introduction

Acute kidney injury (AKI) is defined as decrease in urine production or an increase of retention parameters bringing disturbances in electrolyte, acid-base, and fluid balance [1]. AKI occurs in about 7% of all hospitalized patients and in about 66% [2] of the patients on the ICU. This not only increases mortality but also increases hospital length of stay as well as costs [2,3]. The most frequent reasons for renal impairment are septic shock, surgical intervention, cardiogenic shock, and nephrotoxic medications. About 5 to 6% of these patients require kidney replacement therapy. Uchino *et al*. showed that septic shock (47% of patients) was the most predominant reason for an AKI. Furthermore, they were able to show that in 19% of patients with AKI there was an association with potentially nephrotoxic medications [4]. Treatment of critically ill patients demands a broad therapy regimen of medications, some of them with the potential to seriously damage kidney function either alone or in combination. This includes medications such as aminoglycosides, vancomycin, amphotericin B, angiotensin-converting enzyme inhibitors, diuretics, non-steroidal anti-inflammatory drugs to name only a few [5]. Based on the current literature, there is evidence that 19 to 25% of AKI is influenced by potentially nephrotoxic medication [2-8].

Vancomycin, a glycopeptide antibiotic used in the treatment of methicillin-resistant *Staphylococcus aureus* (MRSA), is accused of causing tubular necrosis when applied in high doses. This mechanism has been demonstrated in animal experiments [9]. Incidence of AKI in humans in relation to vancomycin therapy was demonstrated to occur in 10 to 31% of patients [10,11]. The aminoglycoside antibiotics gentamycin and tobramycin are mostly used in combination therapy against gram-negative pathogens. For these agents, incidence of nephrotoxicity was described in 10 to 20% of patients depending on the dosing regimen [12].

Claims for the adequate anti-infective therapy and preceding diagnostics on ICUs are complex and should be evidence-based. Introduction of guidelines for anti-infective therapy into clinical practice has been shown to improve outcomes on ICU [13,14].

To date, few studies refer to the effect on AKI by using computer-aided decision support for optimizing therapy with potentially nephrotoxic antibiotics. Against this background, we hypothesized that antibiotic therapy following guidelines for nephrotoxic agents is associated with reduced incidence of kidney dysfunction. The primary aim of this study was to investigate the impact of adherence to evidence-based dosing guidelines for potentially nephrotoxic antibiotics on the occurrence of AKI.

## Materials and methods

This observational study was approved by the ethical Committee of the Charité-University Medicine Berlin, Charite Platz 1, 10117 Berlin, and the data safety authorities. The ethics committee waived the need for informed consent from patients due to the observational character of the study. The study was performed as a clinical prospective observational study at the Charité university hospital in Berlin, Germany, a tertiary medical care centre with 3,200 beds. Data collection was performed in two periods from August to October 2009 and from February to April 2010 over a total of 140 days. This study is part of a longitudinal study for the evaluation of the implementation of a stewardship program for antibiotic treatment (ABx Trial Registration: ISRCTN54598675). As this study has a protocol of interrupted time series of 3 months, data were collected according to the protocol.

All patients admitted to the three participating study ICUs during the two study periods were screened for inclusion. The three study-ICUs were led by anaesthesiologists and combined a total of 61 ICU beds. Patient census consisted of mainly postoperative patients from different surgical disciplines (abdominal surgery, cardiac surgery, neurosurgery, trauma surgery and gynaecology) as well as non-surgical patients suffering from acute respiratory distress syndrome (ARDS). Standard operating procedures were the same for the participating ICUs.

Study inclusion criteria were adulthood (≥18 years), ICU treatment of more than 36 h, and medication with one potentially nephrotoxic anti-infective agent (vancomycin, gentamicin, or tobramycin). Patient data were extracted from the patient data management system of the ICU (COPRA GmbH, Sasbachwalden 77887, Germany) as well as from patient files and were collected into electronic case report files (eCRF) covering each treatment day on the ICU. Data on vital signs, laboratory findings, including blood sugar, microbiological and radiological diagnostics, anti-infective, vasopressor and steroid agents, ventilation, pulmonary gas exchange, urine output or dialysis, and fluid balance were obtained.

To ensure consistency, study data were re-evaluated by comparing data from the database with the data from the original patient files in the middle and at the end of each collection period during internal data monitoring. ICU scores, sequential organ failure assessment (SOFA), therapeutic intervention scoring system (TISS), and simplified acute physiology score II (SAPS II), measured regularly on the included ICUs were also documented in the eCRF. Additionally, infection status and the supposed or known focus of infection were documented daily. Infection with a concomitant systemic inflammatory response syndrome was classified as sepsis.

### Study group formation

Adherence was evaluated for every day on therapy. To calculate adherence rates, each treatment day with vancomycin, gentamicin or tobramycin was recorded and assessed. The study day was evaluated as adherent, if all related parts of the guideline were fulfilled. The binary variable of daily adherence, non-adherent versus adherent, was transferred into a relative variable. Therefore the standard operating procedure (SOP) adherence was defined as total number of days with an application of one or more of these antibiotic agents divided by the days on these antibiotics following the guidelines for their application. Evaluation process was performed after closure of the primary database. Evidence-based recommendations were introduced a priori using a computerized decision support program called ABx, which was implemented in 2007. The recommendations included in this programme were comprised of interdisciplinary expert rounds at the Charité hospital. Anti-infective therapy on all study wards is based on this ABx-program which can also be accessed online [15]. Patients were classified into the high-adherence group (HAG) if they had an adherence to SOPs of more than 70% of the treatment days and to the low adherence group (LAG) if they had 70% or fewer adherences to SOPs as described in detail before [16].

The cut off of 70% was selected because this is the quality indicator for ICU implementation rate of our certified institutional quality management according to DIN EN ISO 9001:2000 standard. This cut off was also taken because 70% accounts for an excellent value according to the previous literature and has been published by our working group as having influence on the outcome of ICU patients [16-19].

### SOP evaluation for the therapy with vancomycin

Every consecutive day was evaluated for adherence of SOPs. Vancomycin therapy was separately evaluated for continued and intermittent therapy, each with a specific dosing concept based on renal function and pre-existing kidney damage. For vancomycin initiation in adults, a loading dose of 1 g intravenously is recommended. In the continuous dosing regimen this is followed by 2 g over 24 h (creatinine clearance <50 ml/h with 1 g/24 h, <20 ml/h with 500 mg/24 h). For intermittent dosing, maintenance doses are recommended with 0.5 g/6 h or 1 g/12 h. Regarding therapeutic drug monitoring in continuous therapy, first measurements are obtained after 24 to 36 h with target levels of 15 to 20 mg/l. In intermittent therapy the trough levels are to be obtained before the fourth application of vancomycin with a target level of 10 to 20 mg/l. During maintenance therapy, SOP conformity required adaptation of dosing regimen according to therapeutic drug monitoring.

### SOP evaluation for the therapy with gentamicin and tobramycin

For management of gentamicin and tobramycin therapy, recommendations based on renal function and therapeutic drug monitoring (TDM) were analysed. For initial therapy induction, doses with 3 to 6 mg/Kg body weight in gentamicin and 5 to 7 mg/Kg body weight for tobramycin are used. Maintenance therapy should be adapted to serum trough levels with a 2 mg/l cut off for each agent based on the laboratory reference. The recommendations include adjustment to blood trough levels for the two drugs or dosing adaptation based on elevated serum creatinine levels. Aminoglycosides were administered once daily after achievement of the results of the TDM. Dose adaption was performed according to the results; only if the results were extremely high one dose was omitted. Peak levels have no influence on the dosing and are for this reason not measured regularly.

### Calculation of the adherence rate

To calculate adherence rates, each study day on which a patient was treated with either vancomycin, gentamicin or tobramycin was recorded and evaluated as described in detail elsewhere [16]. For combination therapy all related SOPs had to be fulfilled. The binary variable of daily adherence was transferred into a relative variable. Therefore, in each patient the cumulative number of days with SOP conformity was divided by the total number of days with an application of these antibiotics.

### Primary endpoint - acute kidney injury

AKI was defined following the risk, injury, failure, loss, and end-stage kidney disease (RIFLE) classification [20] of the Acute Dialysis Quality Initiative Group (ADQI) from 2004, stratified as being evaluated and scored. Based on this classification, elevation of serum creatinine, or reduction of urine output are used with the worst criterion as the deciding factor. All study patients were evaluated daily for their serum creatinine level and 24-h urine output and afterwards assigned to RIFLE risk strata. For this study acute kidney failure was defined by the RIFLE R (risk) criteria of 1.5-fold creatinine elevation in relation to basic creatinine level or urine output of <0.5 ml/Kg/h × 6 h. For assessment of basic creatinine level, the creatinine clearance formula for modification of diet in renal disease (MDRD) was used with 75 ml/minute*1.73 m^2^ as the standardized reference clearance [21]. Only episodes of AKI after the beginning of anti-infective therapy with vancomycin, gentamicin or tobramycin were included in this study.

### Statistical analyses

Descriptive population characterization was performed using absolute and relative frequencies in variables with categorical scale level. For variables with continuous variables or values with ordinal scale, means and standard deviation or median and quartiles were compared. Analyses of statistical significance were performed with two-sided alpha <5%. Statistical significance tests were performed with Chi^2^-tests, Student’s *t*-test or Mann-Whitney *U*-test depending on the scale level and distribution of normality. Logistic regression analyses were added to obtain odds ratios (ORs) for covariables and also to define most relevant factors for the dependent variable (occurrence of acute renal injury) using backward selection regression model. To identify a suggestive cut off for SOP adherence rate, primary and secondary end points of the study were evaluated stepwise using different cut offs of SOP adherence. The corresponding LAG and HAG populations for each adherence rate were used to analyse the related significance levels for occurrence of AKI, length of ICU stay, and duration on mechanical ventilation. All analyses were performed using PASW 19.0 (SPSS Inc. 1998-2010, Chicago, Illinois 60606, USA).

## Results

Altogether, 675 patients were screened in both study periods. Finally, the study population included 163 patients undergoing therapy with at least one of the three antibiotics vancomycin, gentamicin or tobramycin. Adherence rate was as follows: vancomycin 75.7% (95% CI 71.8, 79.5), gentamicin 70.3% (95% CI 58.1, 82.4), and tobramycin 69.4% (95% CI 60.0, 78.8); with an overall adherence rate of 73.7% (95% CI 70.0, 77.5). Adherence to SOPs was equal in the two study periods 72.1% (95% CI 66.8, 77.3) in the first and 75.8% (95% CI 70.3, 81.2) in the second period. The LAG consisted of 75 patients (46%), the HAG of 88 patients (54%). Regarding basic characteristics we only observed more soft tissue infections in the LAG than in the HAG (Table 1).

**Table 1:** Basic patient characteristics and distribution of infections

**Characteristics**	**HAG, n = 88 (54%)**	**LAG, n = 75 (46%)**	***P*-value**
Sequential organ failure assessment score (on admission)	6,93 ± 4.85	8.01 ± 4.79	ns
Therapeutic intervention scoring system (on admission)	36.61 ± 11.38	38.97 ± 11.45	ns
Simplified acute physiology score II (on admission)	45.68 ± 19.81	49.81 ± 17.16	ns
Age, years	60.1 ± 15.2	59.8 ± 19.1	ns
Gender, female/male	39/49 (44%/56%)	25/50 (33%/67%)	ns
Immune suppression	11 (13%)	16 (21%)	ns
Comorbidities			
Cardiovascular	53 (60%)	50 (67%)	ns
Chronic pulmonary disease	28 (32%)	26 (35%)	ns
Vascular disease	30 (34%)	37 (49%)	ns
Chronic liver disease	16 (18%)	10 (13%)	ns
Chronic renal disease	31 (35%)	27 (36%)	ns
Metabolic disorders	35 (40%)	32 (43%)	ns
Neurological disease	22 (25%)	19 (25%)	ns
Oncological disease	21 (24%)	15 (20%)	ns
Surgery (before admission)	58 (36%)	49 (30%)	ns
Dialysis (on first day of admission	18 (56%)	14 (44%)	ns
Infections			
Pneumonia	64 (73%)	56 (75%)	ns
Urogenital	11 (13%)	13 (17%)	ns
Central nervous system	11 (13%)	14 (19%)	ns
Abdominal	15 (17%)	10 (13%)	ns
Catheter-associated	23 (26%)	27 (36%)	ns
Soft tissue	8 (9%)	17 (23%)	0.03
Sepsis	72 (82%)	58 (77%)	ns
Septic shock	58 (66%)	52 (69%)	ns

Data are presented as arithmetic mean ± SD or frequencies (%), respectively. HAG, high-adherence group; LAG, low-adherence group; ns, not significant.

**Figure 1:**
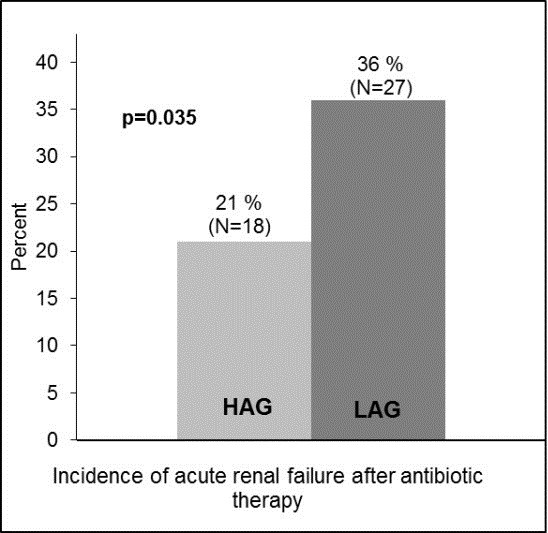
**Incidence of acute renal injury after onset antibiotic therapy compared between study populations.** HAG, patients with high adherence to standards; LAG, patients with low adherence to standards.

AKI occurred significantly more in LAG with 36% versus 21% in HAG (*P* = 0.035) as shown in Figure 1. In the univariate logistic regression as predictors for AKI after antibiotic therapy, the SOFA on admission, chronic kidney failure in the past medical history, immune suppressive therapy, and adherence to SOP showed significant influence (Table 2). Multivariate analysis was performed to further evaluate abovementioned significant factors from univariate analysis and factors derived from the literature. Therefore, variables were included into multivariate logistic regression (SOFA, age, chronic kidney failure, surgery before admission to ICU, septic shock, immune suppression or adherence to SOP). In the last step of the corresponding multivariate regression model, chronic kidney failure was significantly associated with AKI, with an OR of 2.475 (95% CI 1.195, 5.124, *P* = 0.015) as well as SOFA score with an OR of 1.073 (95% CI 0.996, 1.156, *P* = 0.064). In this step, patients in the LAG showed a more than two-fold increased risk of developing AKI with an OR of 2.475 (95% CI 1.195, 5.124, *P* = 0.039; Hosmer-Lemeshow test *χ*^2^ = 8.236; *P* = 0.411, compare Tables 3 and 4).

**Table 2:** Univariate logistic regression of predictors for acute kidney injury

	**Odds ratio (95% CI)**	**P -value**
Age	0.995 (0.976, 1.015)	ns
Gender (female versus male)	1.185 (0.590, 2.384)	ns
ICU scores		
TISS 28 (on admission)	1.024 (0.993, 1.056)	ns
SOFA (on admission)	1.083 (1.008, 1.164)	0.03
SAPS II (on admission)	1.011 (0.992, 1.030)	ns
Comorbidities		
Cardiac	1.629 (0.775, 3.424)	ns
Pulmonary	1.162 (0.564, 2.392)	ns
Vascular disease	0.725 (0.356, 1.475)	ns
Liver	1.201 (0.481, 2.997)	ns
Kidney	2.479 (1.224, 5.020)	0.01
Metabolic disorders	1.370 (0.685, 2.738)	ns
Neurologic disorders	1.116 (0.510, 2.440)	ns
Oncologic disorders	1.424 (0.641, 3.165)	ns
Operation directly before admission		
Abdominal	2.122 (0.865, 5.207)	ns
Cardiac surgery	0.949 (0.428, 2.104)	ns
Septic shock	1.464 (0.684, 3.136)	ns
Infections		
Sepsis	1.534 (0.614, 3.835)	ns
Pneumonia	0.538 (0.255, 1.134)	ns
Urogenital	0.651 (0.228, 1.864)	ns
Central nervous system	1.023 (0.396, 2.645)	ns
Abdominal	1.285 (0.511, 3.226)	ns
Catheter-associated	1.029 (0.489, 2.162)	ns
Soft tissue	1.285 (0.511, 3.226)	ns
Immune suppression	2.497 (1.063, 5.868)	0.04
SOP adherence (LAG versus HAG)	2.187 (1.086, 4.407)	0.023

TISS, therapeutic intervention scoring system; SOFA, sequential organ failure assessment; SAPS II, simplified acute physiology score II; SOP, standard operating procedure; HAG, high-adherence group; LAG, low-adherence group; ns, not significant.

**Table 3:** Multivariate regression analysis including covariates significantly associated with development of AKI in univariate analyses

	**Odds ratio (95% CI)**	***P*-value**
Age	0.984 (0.972, 1.017)	0.607
SOFA (on admission)	1.063 (0.981, 1.151)	0.137
Chronic kidney failure	2.346 (1.061, 5.189)	0.035
Operation before admission	0.907 (0.420, 1.962)	0.805
Septic shock	1.105 (0.472, 2.587)	0.818
Immune suppression	1.621 (0.638, 4.118)	0.310
Adherence to SOP (LAG versus HAG)	2.054 (0.986, 4.280)	0.055

Significant covariates were included in multivariate regression analysis (Hosmer-Lemeshow test, *χ*2 = 10.312; *P* = 0.244). SOFA, sequential organ failure assessment; SOP, standard operating procedure; HAG, high-adherence group; LAG, low-adherence group

**Table 4:** Last step of multivariate regression analysis back-step model including covariates significantly associated with development of AKI in univariate analyses

	**Odds ratio (95% CI)**	***P*-value**
SOFA (on admission)	1.073 (0.996, 1.156)	0.064
Chronic kidney failure	2.475 (1.195, 5.124)	0.015
Adherence to SOP (LAG versus HAG)	2.146 (1.038, 4.437)	0.039

Significant covariates were included in multivariate regression analysis **(**Hosmer-Lemeshow-test, *χ*2 = 8.236; *P* = 0.411). SOFA, sequential organ failure assessment; SOP, standard operating procedure.

**Figure 2:**
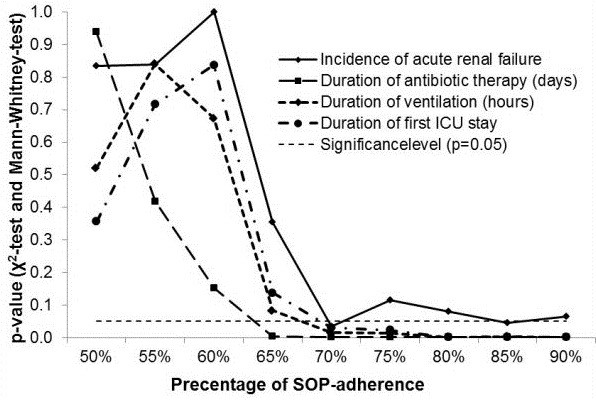
**Stepwise analyses of alpha-value to define standard operating procedure (SOP) cut off for primary and secondary outcome parameters comparing the high-adherence group (HAG) and the low-adherence group (LAG).** HAG, patients with high adherence to standards; LAG, patients with low adherence to standards.

Furthermore, we analysed secondary outcome parameters. Duration of antibiotic therapy was shorter with a median of 5 (25 to 75%, quartile 2 to 8) days in the HAG and 8 (25 to 75%, quartile 4 to 13) days in the LAG (*P* = 0.001). Length of first ICU stay was significantly different between groups with a median of 15 (25 to 75%, quartile 7 to 24) days in the HAG and 22 (25 to 75%, quartile 11 to 31) days in the LAG (*P* = 0.031). The median time of mechanical ventilation was shorter in the HAG with 203 (25 to 75%, quartile 18 to 505) h than in the LAG with 361 (25 to 75%, quartile 104 to 689) h (*P* = 0.018). Mortality did not differ significantly between groups with 15 (17%) patients in the HAG and 14 (19%) in the LAG (*P* = 0.839).To test plausibility for the SOP adherence target score of 70% on the primary and secondary outcome parameter, different break points for SOP adherence were tested stepwise. The results are shown in Figure 2 and are suggestive of a cut off >65 to 70% SOP adherence. In Figure 2 different break points for SOP adherence were tested stepwise and significance levels for SOP adherence are displayed comparing impact on incidence of AKI, duration of treatment with antibiotics, duration of mechanical ventilation, and length of first stay in the ICU.

## Discussion

The most important finding of our study is that AKI was associated with low adherence to SOPs for treatment with gentamicin, vancomycin, and tobramycin. Additionally, the LAG had a prolonged time of mechanical ventilation, a longer first period on ICU, and a longer treatment time with the examined antibiotics. Based on these data, it is suggestive that the degree of compliance influences patients’ outcomes.

Basic characteristics were equally distributed between study groups in relation to gender, age, and comorbidities. For SOFA; SAPS II and TISS-28 on admission, no significant differences were observed. In our population, infections were equally distributed in both groups except soft-tissue infections, while the frequency of infection types was comparable to other studies [22-24]. Furthermore, for the incidence of sepsis, which is an important risk factor for AKI, there was no difference between the HAG and LAG. The 27% incidence of AKI in our population is comparable to previous studies. In a large multinational database Ostermann *et al*. identified impaired renal function based on RIFLE criteriain 36% of more than 40,000 ICU patients [6]. The authors also found ICU scoring systems to be associated with AKI, consistent with our observation in the regression analysis for SOFA score on admission [6]. The increased risk of developing AKI might be explained by the fact that the immune-suppressive agents like calcineurin-inhibitors that are common in our study population have shown inherent nephrotoxicity. For example, the interaction and additive effect of potentially nephrotoxic antibiotics evaluated in this study was shown for the simultaneous application of cyclosporine A and vancomycin [25]. Further investigations focusing on this topic would be needed to evaluate this problem.

The main hypothesis of the study is supported by the results of the univariate analysis demonstrating a clear 15% reduction of AKI incidence between study groups or a more than two-fold increased risk of developing AKI during the ICU-stay based on the regression analysis. These findings suggest that quality of application of vancomycin, gentamicin, and tobramycin influences renal function. Finally, multivariate logistic regression models validated that finding, demonstrating that besides pre-existing chronic kidney failure and poor physiological status in ICU scoring systems, prescribing patterns of nephrotoxic antibiotics have significant influence. Amongst the mentioned factors, adherence to recommendations for antibiotic therapy is the only variable controllable during the ICU stay and inherits the potential to improve morbidity and mortality at least to a certain extent.

Another interesting aspect was the observation, that prolonged application of antibiotics was found in the LAG. Based on the data obtained, it is impossible to differentiate the cause of this finding but there might be an association between dosing regimen and duration of anti-infective therapy. Furthermore, previous studies have demonstrated that with prolonged application of potentially nephrotoxic antibiotic agents, AKI is more likely to occur [26,27]. Following this hypothesis, guideline-adherent therapy might be associated with reduced duration of application and with it the risk of the occurrence of nephrotoxicity, followed by a consecutive decline in kidney function. Additionally, the predictive value of greater adherence to SOPs was shown in preceding studies [13,28]. In our study a high adherence to SOPs was associated with reduced duration of mechanical ventilation and length of stay on the ICU, probably through optimization of antibiotic therapy. In the current observation of mortality, no significant difference between the LAG and HAG was detected. Regarding acute renal insufficiency, morbidity and mortality are linked [29], as occurrence of renal injury is one major determinant of ICU mortality [2].

SOPs exist for various settings in hospital. Implementation of guidelines raises big challenges. For the adherence to our SOPs, we reached an overall level of 73.7%. As 70% is one targeted goal to achieve for implementation [14,17] and was included in our local implementation strategy, the observed implementation rate for vancomycin, gentamicin, and tobramycin seems to be a good value. Additionally, the quality goal of 70% was already successfully adapted for the treatment of pneumonia and is an internationally accepted goal for adherence to SOPs [30]. Previous findings from our group showed that adherence to SOPs for the antibiotic treatment of pneumonia is associated with an improved outcome on ICU [14]. One major difficulty of antibiotic therapy is not only induction but also termination of therapy, especially in patients with renal insufficiency. Prolonged nephrotoxic therapy is one determinant of progressive renal impairment as was demonstrated for vancomycin [31], gentamicin [32], and tobramycin [33]. Comparing different percentages of SOP adherence by the Wilcoxon Mann-Whitney test, there was a significant change in impact on incidence of AKI, duration of treatment with the antibiotics, duration of mechanical ventilation, and length of first stay in the ICU. Figure 2 shows the dependence between duration of treatment of the first pneumonia episode, duration on ventilation, length of stay in the ICU, and percentage of SOP adherence. In our observation all slopes show a concordant transition point at >65% SOP adherence representing significant values for the related variables. This analysis was performed to validate plausibility for the SOP adherence target score of 70% on the primary and secondary end points. The results shown in Figure 2 are suggestive that a cut off >65 to 70% SOP adherence has high implications on clinical outcome.

Obviously, a major limitation is the observational study design because it would need a randomized controlled setting as the gold standard to investigate the hypothesis and to control for hidden confounders. On the other hand, for the adherence to SOPs it is not ethically justifiable, as this design would mean withholding adequate and safe therapy from one study population. Therefore, we carefully assessed for potential confounders. AKI is of multifactorial genesis [4,7] and we only were able to control for a defined set of variables - as treatment with other potentially nephrotoxic medications might influence the results as well. Another limitation could be found in the two treatment periods which took place in different seasons; hence, seasonal effects should not affect the addressed side effects of anti-infective treatment.

## Conclusion

In conclusion, adherence to guidelines for the therapy with potentially nephrotoxic antibiotics is associated with reduced kidney damage. For the adherence goal of 70% it is another brick in the wall towards a desired goal of increased quality in medicine. Further studies are advisable to analyse the validity of the given adherence target in different settings.

## Key messages

•Quality of application of vancomycin, gentamicin, and tobramycin appears to influence renal function.

•High adherence to SOPs may lead to decreased kidney damage when potentially nephrotoxic antibiotics are used.

•An adherence to SOPs above 70% proved beneficial in our population.

•Low adherence to SOPs for antibiotic treatment with potentially nephrotoxic agents in our study was associated with an inferior course in the ICU.

## Abbreviations

ADQI: acute dialysis quality initiative group; AKI: acute kidney injury; ARDS: acute respiratory distress syndrome; eCRF: electronic case report files; HAG: high-adherence group; LAG: low-adherence group; MDRD: modification of diet in renal disease; MRSA: methicillin-resistant *Staphylococcus aureus*; OR: odds ratio; RIFLE: risk injury, failure, loss, and end-stage kidney disease; SAPS II: simplified acute physiology; SOFA: sequential organ failure assessment; SOP: standard operating procedures; TDM: therapeutic drug monitoring; TISS: therapeutic intervention scoring system.

## Competing interest

All authors declare that they have no conflicts of interest concerning the specific subject of this study. IN and ST received lecture fees from Roche Deutschland GmbH and Pfizer Deutschland GmbH. CS received lecture fees or grants for other projects from Abbott, Aspect, Baxter, Deltex, Care Fusion, Fresenius, Hutchinson, Köhler, MSD, MCN, Novartis, Sysmex, Pajunk, Köhler Chemie, Essex Pharm, Pfizer and GSK.

## Authors’ contributions

IN: conception and design, data acquisition, analysis and interpretation of the data, drafting the manuscript. ST: conception and design, data acquisition, analysis and interpretation of the data, drafting of the manuscript. KG: conception of the study, data acquisition, analysis and interpretation of data, critical revision of manuscript. AU: acquisition and interpretation of data, critical revision of the manuscript. RP: acquisition and interpretation of data, critical revision of the manuscript. AT: acquisition and interpretation of data, critical revision of the manuscript. KDW: analysis and interpretation of data, critical revision of the manuscript. CS: conception and design, analysis and interpretation of the data, drafting of the manuscript. All authors had full access to the data, take responsibility for the integrity of the data and the accuracy of the analysis, and have read and approved the final manuscript.

## Authors’ information

Author's affiliation number 1 is the institution where the work was performed.
